# Temporal trends in breast cancer survival by race and ethnicity: A population-based cohort study

**DOI:** 10.1371/journal.pone.0224064

**Published:** 2019-10-24

**Authors:** Deirdre A. Hill, Eric R. Prossnitz, Melanie Royce, Andrea Nibbe

**Affiliations:** 1 Department of Internal Medicine, University of New Mexico, Albuquerque, New Mexico, United States of America; 2 Department of Molecular Medicine, University of New Mexico, Albuquerque, New Mexico, United States of America; Johns Hopkins University School of Medicine, UNITED STATES

## Abstract

**Introduction:**

Differences in breast cancer survival by race and ethnicity are often assumed to be a fairly recent phenomenon, and are hypothesized to have arisen due to gaps in receipt of screening or therapy. The emergence of these differences in calendar time have implications for identification of their origin. We sought to determine whether breast cancer survival differences by race or ethnicity arose in tandem with the advent of screening or therapeutic advances.

**Materials and methods:**

A cohort of women diagnosed with invasive breast cancer from 1975–2009 in 18 population-based registries were followed for five-year breast cancer cause-specific survival. Differences in survival according to race/ethnicity and estrogen receptor status were quantified in Cox proportional hazards models, with estimation of hazard ratios (HR), 95% confidence intervals (CI), and absolute risk differences. For 2010, we also assessed differences in survival by breast cancer subtypes defined by hormone receptor and Her2/neu status.

**Results:**

Among over 930,000 women, initial differences in five-year breast cancer-specific survival by race became apparent among 1975–1979 diagnoses and continued to be evident, with stronger disparities apparent in those of Black vs. White Non-Hispanic (WNH) race and among estrogen-receptor positive vs. negative disease. Within breast cancer subtype, all included race/ethnic groups experienced disparate survival in comparison with WNH women for triple-negative disease. Black women had a consistent gap in absolute survival of .10-.12, compared with WNH women, from 1975–1979 through all included time periods, such that 5- year survival of Black women diagnosed in 2005–09 lagged more than 20 years behind that of WNH women.

**Discussion:**

Survival differed by race for diagnoses that predate the introduction of mammographic screening and most therapeutic advances. Absolute differences in survival by race and ethnicity have remained almost constant over 40 years of observation, suggesting early origins for some contributors.

## Introduction

Breast cancer survival disparities by race and ethnicity have retrospectively been identified using cancer registry and mortality data, with differences in outcome generally believed to have started in the mid-to-late1980’s[1]. Investigators have also sought to understand whether specific events, including the advent of adjuvant endocrine therapy (1990), or taxane chemotherapy (1999)[2] have altered outcomes by race in specific eras, but distinct differences by calendar time have not been apparent. Thus, the emergence of survival differences by race or ethnicity have been attributed in part to increased diffusion of screening mammography since the early 1980’s, and changes in availability and use of adjuvant therapy that began just prior to the decade of the 1990s. As our understanding of the contribution of these sources has grown [2–5], they have accounted for 6% (treatment accounted for 0.8% of the12.9% difference in mortality between whites and blacks)[2] to 25% (screening accounted for 8% and treatment accounted for up to 19% of the mortality difference between whites and blacks)[3] of the increased mortality, while the largest proportion of survival disparities increasingly appears to be attributable to a different source, tumor characteristics at diagnosis (24–66%)[2,5]. However, as the evaluation of survival disparities has occurred primarily in more recent data, earlier data that might hold clues to the origin of survival differences generally have not been explored. Such data may provide insight regarding ultimate sources of outcome divergence if survival disparities arise prior to or in tandem with initiation of new cancer diagnostics or therapeutics.

As early data regarding survival outcomes are an untapped resource, we evaluated temporal patterns in breast cancer survival disparities, including by three race/ethnic groups, in Surveillance Epidemiology End Results (SEER) data beginning in 1975. We sought to identify temporal changes in survival differences, including in tumor marker subsets, that might yield fresh insights regarding their origin.

## Materials and methods

### Study population

We conducted a retrospective analysis of a cohort of incident invasive female breast cancer cases diagnosed in 18 population-based sites of the National Cancer Institute-funded Surveillance Epidemiology End Results (SEER) cancer registry system[6]. Eligible women included those with first invasive breast cancer diagnosed from 1975–2010, and were required to be of White, Black or American Indian/Alaska Native race (the latter includes Aleutian, Alaskan Native or Eskimo, and all indigenous populations of the Western hemisphere). Excluded were breast cancers identified only by autopsy or death certificate, those missing age at diagnosis, and those whose cause of death was unknown. White women of Hispanic ethnicity were included, but Black or American Indian/Alaska Native women of Hispanic ethnicity, who constituted a subgroup too small for analysis, and white women identified only by Hispanic surname were excluded.

### Data collection

SEER utilizes certified tumor registrars to collect information regarding patient demographics, diagnosis, and first course of treatment (generally within four months of diagnosis). Included in this analysis is information ascertained from the medical chart regarding age, sex, race, and Hispanic ethnicity, date of diagnosis, and tumor characteristics (tumor size, lymph node status, and metastasis information). Since 1990, status of the hormone receptors for estrogen and progesterone (positive, negative, borderline, unknown) has also been collected, and in 2010, the tumor marker Her2/neu was added. The three tumor markers allow tumors diagnosed in 2010 to be classified according to four breast cancer subtypes: including hormone receptor positive (HR+), Her2 negative (HER2-) (Luminal A-like), HR+ Her2 positive (Her2+) (Luminal B-like), HR- Her2+ (Her2- overexpressing), and HR- HER2- (Triple Negative). Hispanic ethnicity was determined by the North American Association of Central Cancer Registries (NAACR) Hispanic Identification Algorithm (NHIA) as calculated by SEER. Invasive breast cancer cases were followed for breast cancer-specific death by linkage to local and state vital statistics registries, as well as the National Death Index. Vital status was confirmed by matching to the files of the Centers for Medicare and Medicaid Services. Women were followed until either death, loss-to-follow-up, or December 31, 2014, the end of the study.

### Statistical analysis

Cox proportional hazards models were used to calculate five-year breast cancer-specific survival, with women dying of breast cancer-specific causes within that time period considered “events” and all others censored. Hazard ratios (HR) and 95% confidence intervals (CI) were estimated with an alpha level of .05. Each five-year diagnosis group (i.e., 1990–1994) was followed for a full 60 months (1995–1999), while breast cancer subtype diagnoses in 2010 were followed for up to 60 months (median 51 mo) through end of 2014. All analyses were adjusted for age in ten-year age groups, and for SEER registry start date. Nine registries initiated data collection in 1973–75, four in 1992, and five in 2000. Interaction terms between registry group and race/ethnicity were fit by including the main effects and their product (race/ethnicity * registry group) to determine whether hazard ratios by race differed by registry group, thus suggesting that addition of data from new registries may have altered HRs. Utilizing adjusted 5-year specific survival proportions from Cox models, absolute differences in survival were estimated, and trends with time were quantified using linear regression. Differences in trend by race/ethnicity were evaluated using generalized linear models (GLM). Analyses of estrogen receptor (ER) or progesterone receptor (PR) marker status were restricted to those of known status who were diagnosed in 1990 or later, and breast cancer subtypes to 2010 diagnoses only. The proportional hazards assumption was validated using cumulative sums of Martingale residuals. All analyses were conducted using the SEER Research Data 1975–2014 released in April 2017, based on the November 2016 submission[6]. These SEER research data contain race/ethnicity–specific data since inception (1975 diagnoses), and estrogen receptor data since the 1990 diagnosis year. All analyses were conducted in SAS (v. 9.4, Cary, N.C.). The data analyses were reviewed and approved by the Institutional Review Board of the University of New Mexico Health Sciences Center.

## Results

Overall, 930,910 women diagnosed with invasive breast cancer during 1975–2010 were included in the analysis (S1 Fig), and of those, 128,880 died of breast cancer-related causes during the first five years following diagnosis (13.8%). White Non-Hispanic women comprised the largest proportion (83.5%), followed by black (9.8%), Hispanic white (6.2%), and American Indian/Alaskan Natives (0.5%) (Table 1).

**Table 1:** Characteristics of included women diagnosed with incident invasive breast cancer from 1975–2010 in 18 Surveillance Epidemiology End Results (SEER) registries.

	Cohort		Breast Cancer Deaths	
	N	%	N	%
	n = 930,910		n = 128,880	
**Age:**				
<40	51403	5.5	9744	7.6
40–49	153639	16.5	19519	15.2
50–59	209416	22.5	27750	21.5
60–69	215613	23.2	26729	20.7
70–79	185161	19.9	23969	18.6
≥ 80	115678	12.4	21169	16.4
**Race/Ethnicity**				
White Non-Hispanic	777620	83.5	99873	77.5
Hispanic White	57521	6.2	8287	6.4
Black	91502	9.8	20057	15.6
American Indian/Alaska Native	4267	0.5	663	0.5
**SEER Registry Start Date**				
1973–75	529811	56.9	79573	61.7
1992	110497	11.9	14114	11.0
2000	290602	31.2	35193	27.3
**Estrogen Receptor**	**(n = 870286)**		**(n = 102998)**	
**(Diagnosis** **≥1990)**				
Positive	631080	81.7	46542	58.1
Negative	140972	18.3	33532	41.9
Missing (≥1990)	98234		22924	
**Breast Subtype**	**(n = 52244)**		**(n = 5475)**	
**(Diagnosis 2010** **only)**				
Luminal A-like	33894	73.0	2411	54.5
Luminal B-like	4713	10.1	451	10.2
Her2+ HR-	2048	4.4	362	8.2
Triple Negative	5806	12.5	1202	27.2
Missing (2010)	5783		1049	

Black women had an increased risk of breast cancer specific mortality, relative to White Non-Hispanic white women, beginning in the earliest included time period (1.6-fold) (Table 2). Black women continued to have elevated breast cancer-specific mortality compared to white women for every diagnosis period through the end of follow-up (2.4-fold).

**Table 2:** Race-Ethnicity in relation to five-year breast cancer specific survival by calendar year of diagnosis. Women with Invasive Breast Cancer Diagnosed in 18 SEER Registries, 1975–2009.

Diagnosis Years:	Cohort		Five-Year Breast Cancer Specific Survival	Hazard Ratio 5-year survival (95% CI)
	N	%	N	
**1975–79:**				
White Non-Hispanic	40892	90.9	.76	1.0
Hispanic White	991	2.2	.74	1.0 (0.9–1.2)
Black	3002	6.7	.65	1.6 (1.5–1.7)
American Indian/ Alaska Native	97	0.2	.72	1.3 (0.9–1.9)
**1980–84:**				
White Non-Hispanic	47029	90.0	.78	1.0
Hispanic White	1242	2.4	.75	1.1 (1.0–1.3)
Black	3836	7.4	.66	1.7 (1.6–1.8)
American Indian/ Alaska Native	117	0.2	.71	1.4 (1.0–2.0)
**1985–89:**				
White Non-Hispanic	59746	89.3	.83	1.0
Hispanic White	1940	2.9	.80	1.1 (1.0–1.3)
Black	5038	7.5	.70	1.8 (1.7–2.0)
American Indian/ Alaska Native	206	0.3	.80	1.2 (0.8–1.6)
**1990–94:**				
White Non-Hispanic	76920	85.5	.86	1.0
Hispanic White	4743	5.2	.83	1.2 (1.1–1.4)
Black	7908	8.8	.73	2.1 (2.0–2.2)
American Indian/ Alaska Native	429	0.5	.81	1.4 (1.1–1.8)
**1995–99:**				
White Non-Hispanic	93879	84.0	.88	1.0
Hispanic White	6536	5.9	.83	1.6 (1.4–1.8)
Black	10544	9.5	.76	2.1 (2.0–2.2)
American Indian/ Alaska Native	637	0.6	.82	1.9 (1.6–2.4)
**2000–04:**				
White Non-Hispanic	201527	82.5	.88	1.0
Hispanic White	16057	6.6	.85	1.5 (1.3–1.6)
Black	25235	10.4	.77	2.3 (2.2–2.4)
American Indian/ Alaska Native	1113	0.5	.86	1.6 (1.3–2.0)
**2005–09:**				
White Non-Hispanic	202216	79.8	.89	1.0
Hispanic White	21112	8.3	.87	1.3 (1.2–1.5)
Black	28942	11.4	.80	2.4 (2.2–2.5)
American Indian/ Alaska Native	1340	0.5	.86	1.7 (1.3–2.3)

Hispanic women had a significantly elevated risk of breast cancer-specific mortality beginning with diagnoses in 1990–1994 (1.2-fold), relative to White Non-Hispanic women, and risk remained elevated through 2005–2009 diagnoses (Table 2). While the hazard ratio for mortality appeared to peak in 1995–1999, the hazard ratio for those years and subsequent five-year time periods did not statistically differ from one another.

For American Indian/Alaskan Native women, breast cancer-specific mortality was elevated 1.4-fold compared to White Non-Hispanic women for diagnoses in 1980–1984, and again for 1990–1994 diagnoses, then American Indian/Alaskan Native women continued to have an elevated hazard ratio through the end of follow up in 2014 (2005–2009 diagnoses) (Table 2). Similar to Hispanic women, hazard ratios for mortality peaked in 1995–1999 then declined slightly, although more recent hazard ratio estimates did not differ significantly from those in the 1995–1999 diagnosis years.

**Fig 1:**
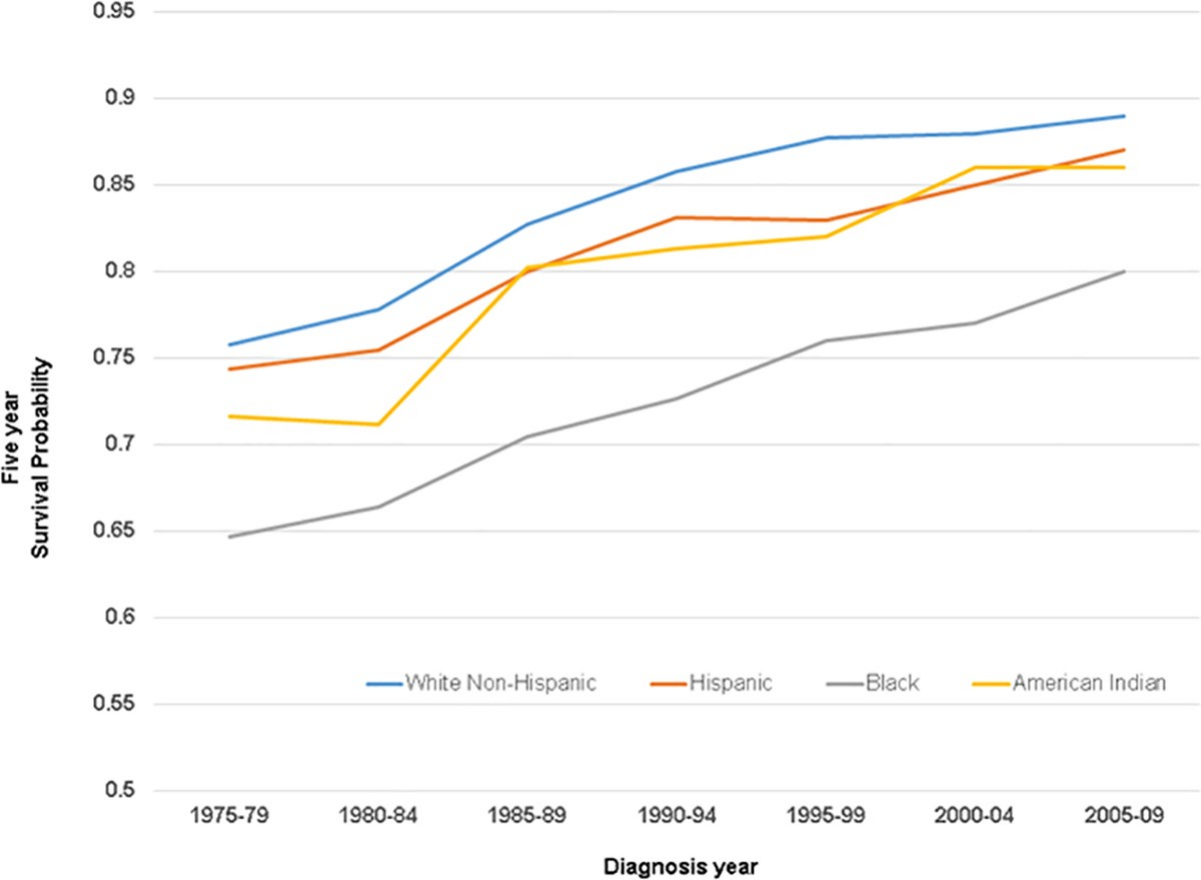
Five-year breast cancer-specific survival by race and ethnicity. 1975–1979 through 2004–2009 diagnoses.

Five-year breast cancer-specific absolute survival estimates increased with time for all race/ethnic groups (Fig 1). Notably, an absolute difference of approximately 12 percentage points between Black and White Non-Hispanic women persisted throughout most of the observation period, such that Black women diagnosed in 2005–2009 (.80) (most recent period with 5-year survival) had attained the survival experienced by White Non-Hispanic women after 1980–84 diagnoses (an over 20-year lag, or 22 years when modeled by exact year). Similarly, Hispanic (11 years) and American Indian/Alaska Native (15 years) absolute survival lagged by over a decade behind that of White Non-Hispanic women.

**Fig 2:**
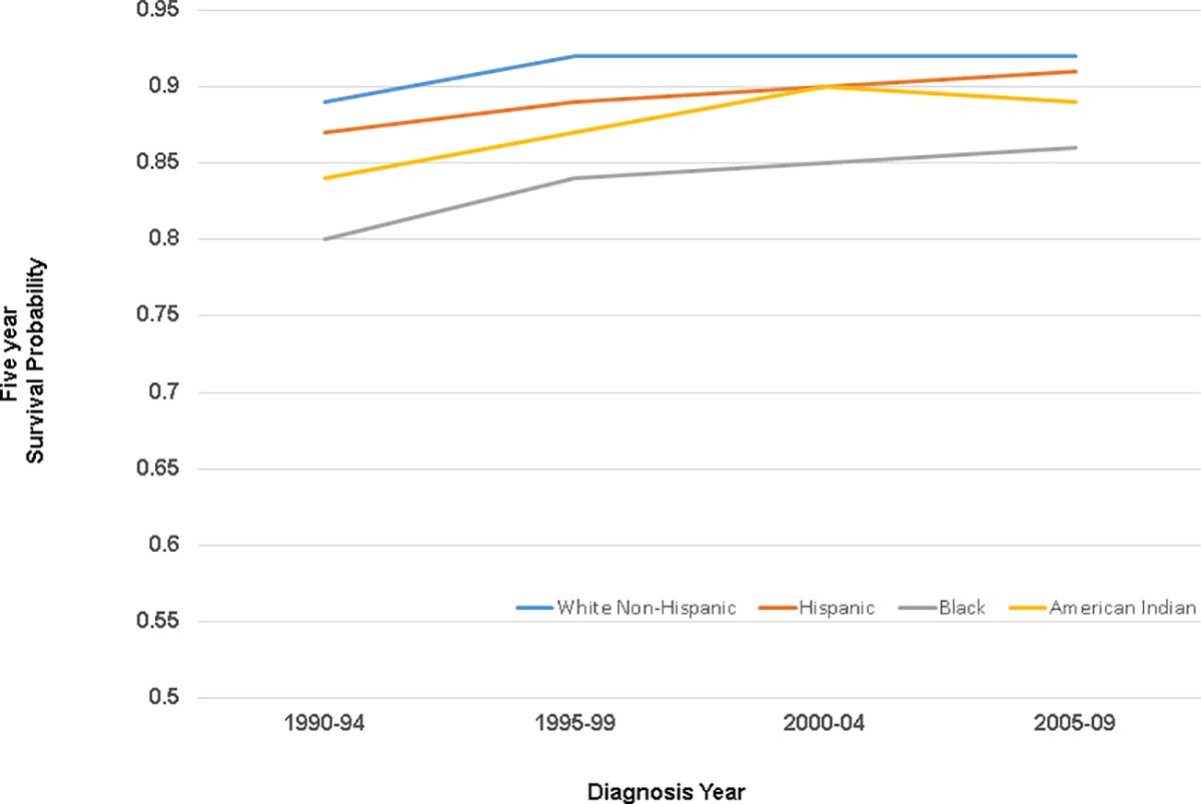
Estrogen receptor positive women: Five-year breast cancer-specific survival by race and ethnicity, 1990–94 through 2004–2009 diagnoses.

Collection of estrogen receptor (ER) information began with 1990 diagnoses (Table 3). Among ER positive women, Black, Hispanic and American Indian/Alaskan Native women all had an increased risk of dying of breast cancer-related causes (1.2–2.4-fold) relative to White Non-Hispanic women in 1990–1994 diagnoses, which continued through end of follow-up. Five-year breast cancer -specific survival estimates ranged from .80 (Black women) in 1990–1994 to .92 (White Non-Hispanic women) in 2005–2009, with race/ethnic group survival estimates generally differing by 4 to 8 percentage points from White Non-Hispanics in each time period (Fig 2). Women with ER positive disease appeared to have a greater breast cancer survival disparity than their counterparts with ER negative disease (p-value for interaction = .0001 for Black, .006 for Hispanic, and .32 for American Indian/Alaska Native, relative to White Non-Hispanic women).

**Table 3:** Race and ethnicity in relation to breast cancer specific survival by estrogen receptor status and five-year diagnosis group. Women with Invasive Breast Cancer Diagnosed in 18 SEER Registries, 1975–2009.

	Estrogen Receptor Positive				Estrogen Receptor Negative			
Diagnosis Years:	Cohort		Five-Year Breast Cancer Specific Survival*	Hazard Ratio (95% Confidence Interval)	Cohort		Five-Year Breast Cancer Specific Survival*	Hazard Ratio (95% Confidence Interval)
	N	%			N	%		
**1990–94:**								
White Non-Hispanic	44507	88.6	.89	1.0	12954	79.7	.77	1.0
Hispanic White	2261	4.5	.87	1.3 (1.1–1.4)	1027	6.3	.75	1.1 (1.0–1.3)
Black	3244	6.5	.80	2.0 (1.8–2.2)	2190	13.5	.67	1.6 (1.4–1.7)
American Indian/ Alaska Native	206	0.4	.84	1.6 (1.1–2.4)	90	0.5	.75	1.2 (0.8–1.8)
**1995–99:**								
White Non-Hispanic	60132	87.8	.92	1.0	15726	78.0	.78	1.0
Hispanic White	3296	4.8	.89	1.4 (1.3–1.6)	1406	7.0	.73	1.3 (1.2–1.5)
Black	4723	6.9	.84	2.1 (2.0–2.3)	2861	14.2	.69	1.5 (1.4–1.7)
American Indian/ Alaska Native	344	0.5	.87	1.7 (1.3–2.3)	157	0.8	.67	1.7 (1.3–2.2)
**2000–04:**								
White Non-Hispanic	133662	85.7	.92	1.0	32684	74.4	.79	1.0
Hispanic White	9173	5.9	.90	1.4 (1.3–1.5)	3599	8.2	.75	1.2 (1.1–1.3)
Black	12288	7.9	.85	2.1 (2.0–2.2)	7423	16.9	.69	1.6 (1.5–1.7)
American Indian/ Alaska Native	715	0.5	.90	1.4 (1.1–1.8)	236	0.5	.76	1.2 (0.9–1.6)
**2005–09:**								
White Non-Hispanic	152926	82.5	.92	1.0	32680	70.4	.80	1.0
Hispanic White	14305	7.7	.91	1.3 (1.2–1.4)	5021	10.2	.78	1.2 (1.1–1.3)
Black	17198	9.3	.86	2.0 (1.9–2.1)	9237	18.8	.73	1.5 (1.4–1.6)
American Indian/ Alaska Native	946	0.5	.89	1.6 (1.3–1.9)	292	0.6	.75	1.4 (1.1–1.8)

**Fig 3:**
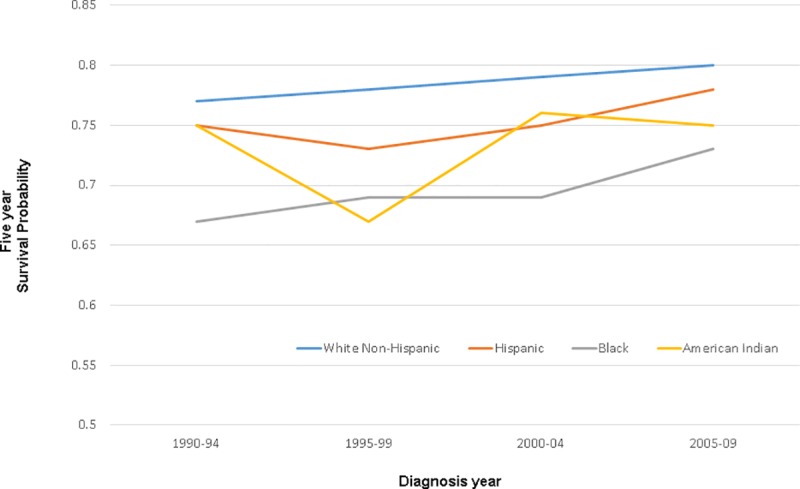
Estrogen receptor negative women: Five-year breast cancer-specific survival by race and ethnicity, 1990–94 through 2004–2009 diagnoses.

Among women with ER-negative tumors, breast cancer-specific survival was also lower among Hispanic, Black, and American Indian/Alaskan Natives in all time periods compared to White Non-Hispanics (Table 3). Breast cancer-specific survival estimates for 5-year increments ranged from .67 (Black women in 1990–1994 and American Indian/Alaskan Native women in 1995–1999 diagnoses) to .80 (White Non-Hispanic women for 2005–2009 diagnoses). Absolute differences in survival proportion exhibited more variability among women with ER negative disease, reflecting the smaller sample size available despite the inclusion of five-year diagnosis groups, with differences between White Non-Hispanic and other race/ethnic groups ranging from 2 to 11 percentage points by time periods (Fig 3).

The proportional hazards assumption did not hold for a few diagnosis years or race-ethnic subgroup analyses overall or in ER-positive or ER-negative analyses. Specifically, violation of the assumption only occurred in 1990 or later diagnoses, when the initiation of data collection in new SEER registries caused some subgroups to double in size, thus facilitating the detection of seemingly small quantitative differences. Survival results are split into 1–24 and 25–60 months post-diagnosis for comparison (Table 4 –assumption violations in bold) although a difference across only a few months sometimes appeared to prompt the violation. Absolute risk estimates for 5-year breast cancer-specific survival do not require the proportional hazards assumption.

**Table 4:** Comparison of hazard ratio estimates for breast cancer survival in months 1–24 and 25–60 post diagnosis. Relationships that failed to meet the proportional hazards assumption denoted in bold and by **. All others (non-bold) provided for completeness.

Diagnosis Years	1–24 mo post diagnosis	25–60 mo post diagnosis
	Hazard ratio 95% CI^	Hazard ratio 95% CI^
**1990–1994:**		
White Non-Hispanic	1.0	1.0
Hispanic**	**1.42 (1.22–1.57)**	**1.30 (1.18–1.44)**
Black	2.36 (2.21–2.52)	1.90 (1.78–2.04)
American Indian/ Alaskan Native	1.14 (0.79–1.65)	1.29 (0.95–1.77)
**1995–1999:**		
White Non-Hispanic	1.0	1.0
Hispanic	1.57 (1.42–1.72)	1.48 (1.36–1.62)
Black**	**2.36 (2.22–2.51)**	**1.90 (1.78–2.03)**
American Indian/ Alaskan Native	1.57 (1.19–2.08)	1.71 (1.33–2.20)
**2000–2004:**		
White Non-Hispanic	1.0	1.0
Hispanic**	**1.51 (1.42–1.61)**	**1.38 (1.30–1.47)**
Black	2.51 (2.41–2.62)	1.97 (1.89–2.06)
**American Indian/** Alaskan Native**	**1.10 (0.84–1.44)**	**1.57 (1.28–1.93)**
**2005–2009:**		
White Non-Hispanic	1.0	1.0
Hispanic**	**1.33 (1.25–1.41)**	**1.30 (1.23–1.38)**
Black	2.25 (2.16–2.35)	1.89 (1.80–1.97)
**American Indian/** Alaskan Native**	**1.49 (1.20–1.85)**	**1.52 (1.24–1.85)**
**Estrogen receptor positive:**		
**Diagnosis Years**	**1–24 mo post diagnosis**	**25–60 mo post diagnosis**
	Hazard ratio 95% CI^	Hazard ratio 95% CI^
**1990–1994:**		
White Non-Hispanic	1.0	1.0
Hispanic	1.39 (1.14–1.69)	1.25 (1.07–1.47)
Black	2.34 (2.05–2.67)	1.83 (1.63–2.05)
American Indian/ Alaskan Native	1.26 (0.66–2.42)	1.54 (0.98–2.42)
**1995–1999:**		
White Non-Hispanic	1.0	1.0
Hispanic	1.34 (1.10–1.63)	1.48 (1.28–1.69)
Black**	**2.56 (2.26–2.89)**	**1.92 (1.73–2.14)**
American Indian/ Alaskan Native	1.81 (1.09–3.00)	1.70 (1.16–2.49)
**2000–2004:**		
White Non-Hispanic	1.0	1.0
Hispanic	1.37 (1.21–1.54)	1.37 (1.25–1.50)
Black**	**2.49 (2.30–2.69)**	**1.92 (1.79–2.06)**
American Indian/** Alaskan Native	1.06 (0.66–1.71)	1.63 (1.22–2.18)
**2005–2009:**		
White Non-Hispanic	1.0	1.0
Hispanic**	**1.20 (1.08–1.32)**	**1.28 (1.18–1.39)**
Black	2.10 (1.96–2.25)	1.97 (1.86–2.10)
American Indian/ Alaskan Native	1.74 (1.27–2.37)	1.51 (1.15–1.96)
**Estrogen receptor negative:**		
**Diagnosis Years**	**1–24 mo post diagnosis**	**25–60 mo post diagnosis**
	Hazard ratio 95% CI^	Hazard ratio 95% CI^
**1990–1994:**		
White Non-Hispanic	1.0	1.0
Hispanic	1.14 (0.94–1.38)	1.11 (0.91–1.34)
Black	1.63 (1.45–1.84)	1.51 (1.34–1.71)
American Indian/ Alaskan Native	1.09 (0.59–2.04)	1.28 (0.73–2.26)
**1995–1999:**		
White Non-Hispanic	1.0	1.0
Hispanic	1.29 (1.11–1.51)	1.37 (1.16–1.61)
Black	1.57 (1.42–1.75)	1.50 (1.34–1.69)
American Indian/ Alaskan Native	1.59 (1.07–2.36)	1.83 (1.22–2.74)
**2000–2004:**		
White Non-Hispanic	1.0	1.0
Hispanic	1.25 (1.13–1.39)	1.23 (1.10–1.36)
Black**	**1.74 (1.63–1.86)**	**1.45 (1.35–1.56)**
**American Indian/** Alaskan Native**	**0.85 (0.54–1.31)**	**1.60 (1.14–2.24)**
**2005–2009:**		
White Non-Hispanic	1.0	1.0
Hispanic	1.17 (1.07–1.29)	1.12 (1.01–1.24)
Black**	**1.67 (1.57–1.78)**	**1.35 (1.25–1.45)**
American Indian/ Alaskan Native	1.33 (0.95–1.86)	1.48 (1.04–2.09)

Abbreviations: ^ CI–confidence interval

** Proportional hazards assumption violated

Note: Proportional hazards assumption violated only in 1990–1994 diagnoses or later.

To determine whether addition of data from new SEER registries influenced these results, an interaction term for grouped registry start data (1973–1975, 1992, 2000) and race/ethnicity was fit. For 1995–1999 and later years, survival was significantly greater than expected on a multiplicative scale for American Indian/Alaskan Natives in registries that initiated data collection in 1992, suggesting that addition of new SEER registries may have increased survival estimates for American Indian/Alaska Native women (data not shown).

Five-year survival by race/ethnicity increased consistently throughout the observation period for White Non-Hispanic women (linear slope = .023; p-value for trend .001) (a slope of .02 suggests an increase in survival such as from .85 to .87 for each 5-year follow up period), Hispanic women (slope = .021; p-trend < .0001; Black women (slope = .026, p-trend < .0001), and American Indian/Alaska Native women (slope = .028; p-trend = .004). Slope did not differ by race-ethnic group (p-value = .68). Over the entire period of observation, 5-year survival for White Non-Hispanic and Hispanic women each equally increased by .13 (from .74 to .87 for Hispanic women for instance), while American Indian/Alaska Native women gained .14 (from .72 to .86) and Black women gained .15 (from .65 to .80). ER-specific slopes were not estimated because statistically stable estimates required more than four time points.

Women diagnosed in 2010 and followed through the end of 2014 offered an opportunity to evaluate five-year breast cancer-specific survival by breast subtype (Table 5). Black and Hispanic women with Luminal A or Luminal B-like disease experienced increased mortality (1.3–2.1-fold), relative to White Non-Hispanic women with the same subtypes. Among women with Her2 overexpressing tumors, only Black women had a greater risk of dying of breast cancer related causes (1.4-fold) than White Non-Hispanic women. Black, Hispanic and American Indian/Alaskan Native women with triple negative tumors all were more likely to die of their disease (1.3–2.3-fold) than White Non-Hispanic women with that subtype.

**Table 5:** Breast cancer subtypes in relation to breast cancer-specific mortality, by race-ethnicity. Women diagnosed in 2010 only, with up to 5 years of follow-up.

	Cohort		Five-year BCSS	Hazard Ratio (95% CI)
	N	%		
**Luminal A-like**	**N = 33894**			
White Non-Hispanic	27747	81.8	.93	1.0
Hispanic	2674	7.9	.90	1.4 (1.2–1.6)
Black	3286	9.7	.85	2.1 (1.9–2.4)
American Indian	187	0.6	.90	1.4 (0.9–2.4)
/Alaskan Native				
**Luminal B-like**	**N = 4713**			
White Non-Hispanic	3560	75.5	.89	1.0
Hispanic	488	10.4	.87	1.4 (1.0–1.9)
Black	632	13.4	.83	1.8 (1.4–2.2)
American Indian	33	0.7	.87	1.4 (0.5–3.8)
/Alaskan Native				
**HER2+ overexpressing**	**N = 2048**			
White Non-Hispanic	1463	71.4	.81	1.0
Hispanic	237	11.6	.80	1.1 (0.8–1.5)
Black	334	16.3	.74	1.4 (1.1–1.9)
American Indian	14	0.7	.83	0.9 (0.2–3.5)
/Alaskan Native				
**Triple Negative**	**N = 5806**			
White Non-Hispanic	3934	67.8	.79	1.0
Hispanic	605	10.4	.75	1.2 (1.0–1.5)
Black	1233	21.2	.71	1.5 (1.3–1.7)
American Indian	34	0.6	.59	2.3 (1.3–4.1)
/Alaskan Native				
Missing Subtype	5783			

## Discussion

Our findings provide a broad perspective on the development of breast cancer survival disparities by race/ethnicity. Our results suggest that gaps in survival by race are present even in the earliest available SEER data (1975–1979), predating breast cancer screening and innovations in adjuvant therapeutics. Thus, screening and therapeutic advances alone are unlikely to entirely account for survival differences by race/ethnicity. Some results are novel, including five-year breast cancer-specific absolute survival estimates by race/ethnicity, linear trends for 1975–2009 diagnoses, recognition of a constant absolute survival difference of 10–12 points between Black and White Non-Hispanic women across the observation period, manifested as a 22 year lag in Black women’s survival attainment, hazard ratio estimates for diagnosis years prior to 1980, and statistically significant differences in breast cancer mortality among Black vs White Non-Hispanic women in Her2-overexpressing tumor subtypes, and that for all race/ethnic subgroups vs. White Non-Hispanic in triple-negative disease. Breast cancer-specific survival estimates by diagnosis year and tumor marker for American Indian/Alaskan Natives have not previously been reported, to our knowledge. Survival trends by race/ethnicity illustrate that absolute increases over the 1975–2014 period were similar for each group of women, but the baseline survival (1975–1979) differed substantially, suggesting the nature of the gap that needs to be closed.

Our findings should be evaluated with consideration of the strengths and limitations of the study. SEER registry staff collect race and ethnicity as indicated in medical records, and do not have independent means to verify recorded information. Similarly, while tumor marker classification may be derived from pathology reports, some may be abstracted from medical charts. The definition of ER and PR positivity changed from 10% to 1% positive staining in 2010 [7], and retrospective information that would allow results to be standardized to the current definition is not available. In addition, despite the inclusion of data from 18 SEER registries and 40 years of diagnoses, sample sizes were limited for some estimates, including for American Indian/Alaskan Natives and rarer breast cancer subtypes.

When examined across 5-year diagnosis periods, breast cancer survival estimates by race/ethnicity present several contrasting observations. Since 1990–1994 diagnoses, gaps in survival on the hazard ratio scale have become evident for Hispanic and American Indian/Alaskan Natives. One potential explanation is increasingly accurate reporting of race and ethnicity with time. Access to advances in healthcare may also be implicated. However, in 1992, additional SEER registries initiated data collection, leading to over a doubling in numbers of breast cancer cases in those race/ethnic groups compared to 1985–89 (Table 2). While our analyses suggest that hazard ratios are homogeneous in both registry groups, the additional cases may have allowed detection of smaller but statistically significant differences.

Although the relative hazard estimates suggest an increase over time in survival disparities for Black women, an absolute risk difference of 10 to 12 percent in comparison with White Non-Hispanic women has been fairly constant for each 5-year observation period. Hazard ratio increases are apparent because the 12-point gap is measured against a White Non-Hispanic (reference group) approaching over 90% 5-year survival with time. While newly arisen factors may have contributed to the 12-point gap to a varying extent over the observation period, rather than a persistent contribution from baseline factors present in 1975–1979, the study data cannot distinguish between these hypotheses. The overall improvements in breast cancer-specific mortality, coupled with a lag, possibly of over 20 years in length, in attaining equivalent absolute survival for some presents a distinct challenge to policymakers, health care providers, and affected women.

ER positive disease currently allows a wider range of treatment options and confers a more favorable prognosis. Thus, the findings raise questions regarding the source of the higher HR for race/ethnic disparities evident in less aggressive disease. Our results are consistent with those in other investigations [5,8–11] including several in which survival differences by race/ethnicity (on a multiplicative scale) are stronger among women with ER-positive disease. While endocrine therapy adherence for five or more years may only be attained by a fraction of those treated [12,13] and thus would constitute a compelling explanation for poor survival in this subgroup, Black or Hispanic women have had reduced adherence to such therapy in some previous studies [14,15] butt adherence equivalent to White Non-Hispanic women in others [16].

Breast cancer subtype-specific survival information should further inform efforts to close survival gaps. Her2-overexpressing disease, not previously identified as subject to differential outcomes by race/ethnicity, may warrant attention directed to appropriate therapy receipt and adherence as in other subtypes. In several studies, race/ethnic women have had poorer outcomes despite either receipt of guideline-based treatments [17], or standard of care in clinical trials [8,18,19]. Thus, hitherto unrecognized tumor biology[20] differences by subgroup may be driving some disparate prognosis. Our results hint that a multi-pronged approach, uniting access to appropriate care and support for adherence, with more precise tumor-specific therapeutic options may pave the way to narrow and ultimately eliminate survival differences.

## Supporting information

S1 Fig Flow diagram: Inclusion criteria for breast cancer cases. (TIF)
