# Supplementary figures and images for: Temporal trends in breast cancer survival by race and ethnicity: A population-based cohort study
Source: PLoS One. 2019 Oct 24;14(10):e0224064. doi: 10.1371/journal.pone.0224064 (PMC6812853; doi:10.1371/journal.pone.0224064)

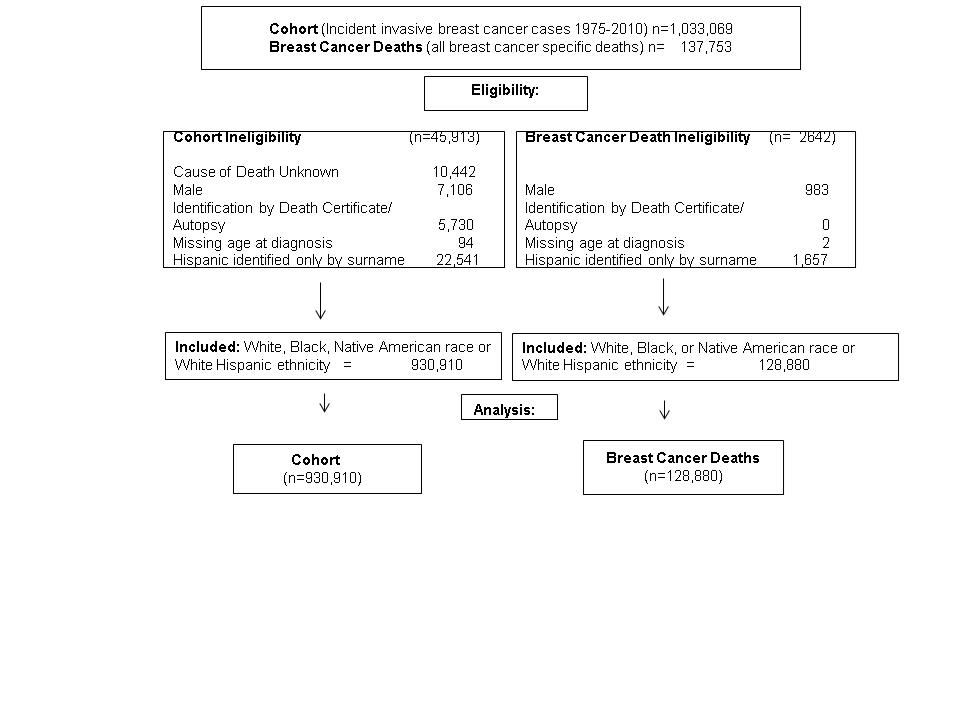

Supplement: S1 Fig — (TIF) [file pone.0224064.s001.tif]
